# Effects of dietary Selenomethionine supplementation on growth performance, antioxidant status, plasma selenium concentration, and immune function in weaning pigs

**DOI:** 10.1186/2049-1891-5-46

**Published:** 2014-10-02

**Authors:** Jun Cao, Fucun Guo, Liying Zhang, Bing Dong, Limin Gong

**Affiliations:** State Key Laboratory of Animal Nutrition, Ministry of Agriculture Feed Industry Centre, China Agricultural University, Beijing, 100193 China; Novus International (Beijing) R & D Center, Beijing, 100085 China

**Keywords:** Antioxidant status, Growth performance, Plasma selenium concentration, Selenomethionine, Weaning pigs

## Abstract

**Background:**

This study was designed to evaluate the efficacy of DL-selenomethionine (DL-SeMet) supplementation on growth performance, antioxidant status, plasma selenium (Se) concentration, and immune function of weaning pigs. 216 weaning pigs were randomly allocated to 6 treatments with 6 replicates each according to a complete randomized block design. Each replicate had six pigs. Diet of group one was corn-soybean basal diet without any additional Se supplement. Group 2 was supplemented with 0.3 mg/kg of Se from sodium selenite. Groups 3-6 were supplemented with 0.1, 0.3, 0.5, and 0.7 mg/kg of Se from DL-SeMet, respectively. The trial lasted for 42 days.

**Results:**

Pigs supplemented with 0.3 and 0.7 mg/kg DL-SeMet obtained better feed gain ratio (*P* < 0.05). The best antioxidant ability (serum, liver, and muscle) was shown in 0.1-0.3 mg/kg DL-SeMet groups (*P* < 0.05). The plasma Se concentration increased as the dietary DL-SeMet level elevated. The immunity among groups was not affected.

**Conclusions:**

DL-SeMet supplementation in the diet significantly improved the growth performance, antioxidant ability and plasma Se content of weaning pigs. DL-SeMet can replace sodium selenite in the diet of weaning pigs.

## Background

As an essential trace element, selenium (Se) can enhance immunity, anti-oxidation, and anti-cancer capacity of human and animals. The deficiency of Se may lead to muscular dystrophy, exudative diathesis, necrotic liver degeneration, and mulberry heart disease in farm animals [1]. On the other hand, high dose of Se can cause selenosis in swine involving hair loss, cracking of hooves, and an interruption in coronary band development of the hoof [2, 3]. In the animal industry, it is generally believed that widely used inorganic Se additives (such as sodium selenite) are cheap but highly toxic, while organic Se is considered more efficient in absorption, anti-oxidation, and tissue accumulation, less toxic and environmentally pollutional as a dietary supplementation [4–6]. Accordingly, different sources of organic Se additives have been commercialized.

For the past few years, Se-enriched yeast attracted a lot of attention in animal nutrition. Many researches indicated that Se-enriched yeast was very effective in increasing growth performance, glutathione peroxidase (GSH-Px) activity, tissue Se concentration, and improving carcass quality of broilers or growing-finishing pigs [7–10]. In addition, Peretz et al. [11] demonstrated that Se-enriched yeast showed immunostimulatory properties in elderly humans. Considering that selenomethionine has been recognized as the major form of organic Se in Se-enriched yeast [12], we proposed that selenomethionine could function more efficiently than Se-enriched yeast compounds.

Until recently, only a few studies concluded that maternal selenomethionine supplementation improved growth performance and Se deposition of offspring more efficiently than sodium selenite supplementation [13, 14]. Some research results showed that selenomethionine could improve anti-oxidative capacity and meat quality of broilers or finishing pigs [15, 16]. Some researchers were focused on the maximum additive amount of Se to the diet of domestic animals [17–19]. The NRC (2012) [20] recommendations established a level of 0.15-0.30 mg/kg of Se supplementation for pigs in different body weight. Maximum level of Se (from sodium selenite or Se-enriched yeast) in complete feeds has been set at 0.5 mg/kg in China [21] and European Union [22, 23] to ensure feed safety. But there’s few study about the effect of adding DL-selenomethionine (DL-SeMet) in weaning pig diets in China and elsewhere. This is the first study focused on the effect of DL-SeMet on weaning pigs. Meanwhile, DL-SeMet is not yet approved to be added to feedstuffs in China [24].

Therefore, the objective of this study was to evaluate the efficacy of DL-SeMet in weaning pig diets. The experiment was conducted to investigate the effects of different dietary levels (0.1-0.7 mg/kg) of DL-SeMet supplementation on growth performance, antioxidant status, plasma Se concentration and serum immunoglobulin levels in weaning pigs.

## Materials and methods

The protocol for the animal experiment was reviewed and approved by the Institutional Animal Care and Use Committee at China Agricultural University.

### Se sources

In this study, two types of Se were applied. The DL-SeMet, as an organic Se source and containing 2,000 mg/kg Se, was provided by NOVUS Intl. (MO, USA). While the 0.5% sodium selenite premix was provided by Tongli Xingke Agricultural Science and Technology Co., Ltd (Beijing, China).

### Animals and experimental design

A total of 216 crossbred (Duroc × Landrace × Yorkshire) weaning pigs (weaned on d 28, initial body weight 9.94 ± 1.26 kg) were randomly allocated to 6 treatment groups with 6 replicates each (one pen per replicate) according to a complete randomized block design. Each replicate contained six pigs with male and female evenly divided. Diet of group one was corn-soybean basal diet without any additional Se supplement (also named as low-Se group). Group 2 was supplemented with 0.3 mg/kg of Se from sodium selenite, groups 3-6 were supplemented with 0.1, 0.3, 0.5, and 0.7 mg/kg of Se from DL-SeMet, respectively (as-fed basis). The measured values of Se in diet 1-6 were 0.06, 0.35, 0.44, 0.51, 0.57 and 0.99 mg/kg, respectively. The feeding trial lasted for 42 days. Sodium selenite and DL-SeMet were added to the diets at the expense of corn. Before the start, all weaning pigs were fed the basal diet for one week as pretreatment.

The corn-soybean meal basal diet was formulated to meet the nutrient requirements according to NRC (2012) [20] except Se (Table 1). Sodium selenite and DL-SeMet were premixed in corn then added to each diet.

**Table 1:** **Ingredients and nutrient content of the basal diet (%, as-fed basis)**

Ingredients	%	Nutrient content ^2^	%
Corn	58.56	Digestible energy, kcal/kg	3492.98
Soybean meal (46% CP)	14.00	Crude protein	18.87
Soybean oil	1.60	Calcium	0.70
Extruded-soybean	12.00	ATTDP ^3^	0.29
Fish meal	3.45	Lysine	1.40
Whey powder (12% CP)	7.00	Methionine	0.48
Calcium hydrophosphate	1.08	Methionine + cysteine	0.79
Limestone	0.60	Threonine	0.88
Sodium chloride	0.32	Se, mg/kg	0.06
L-Lysine hydrochloride	0.43		
Threonine	0.12		
DL-Methionine (99%)	0.14		
Choline chloride (50%)	0.20		
Vitamin-mineral premix^1^	0.50		
Total	100.00		

^1^Provided per kilogram of diet: vitamin A 11,000 IU, vitamin D_3_ 1,500 IU, vitamin E 44.1 IU, vitamin K_3_ 4.0 mg, vitamin B_1_ 1.4 mg, vitamin B_2_ 5.22 mg, vitamin B_5_ 20.0 mg, vitamin B_12_ 0.01 mg, niacin 26.0 mg, pantothenic acid 14 mg, folic acid 0.8 mg, biotin 44 μg, Fe 100.0 mg, Cu 16.50 mg, Zn 90.0 mg, Mn 35.0 mg, I 0.30 mg.

^2^Crude protein and calcium were measured value, others were calculated.

^3^ATTDP: apparent total tract digestible phosphorus.

This experiment was conducted in the Fengning Experimental Base (China Agricultural University, Beijing, China). Temperature in the pigpen was maintained at about 25°C. The pigs had ad libitum access to diet and water. Health condition, feed consumption, and mortality of pigs in each replicate were recorded every day. At the beginning (d 0), middle (d 21), and end (d 42) of the experiment, pigs in 6 treatments were deprived of feed for 12 h then weighed per replicate. Average daily gain (ADG), average daily feed intake (ADFI), and feed gain ratio (F:G) were calculated.

### Sampling and processing

At the middle and end of the experiment, after deprived of feed for 12 h, one pig per replicate (always the same pig of each replicate) was selected to collect two blood samples from precava. One of them was anticoagulant (each tube contained 10 mg ethylenediamine tetraacetic acid dipotassium salt as anticoagulant) blood sample, from which plasma was separated by centrifugation at 4°C, 3,000 rpm for 10 min and transferred into 1.5 mL micro centrifuge tubes. The other was without anticoagulant and coagulated at room temperature for 1 h. Serum was separated by centrifugation and also transferred into 1.5 mL micro centrifuge tubes. At the end of the experiment, the same 36 chosen pigs were killed after blood collection. Samples of livers and breast muscles were collected and chilled in liquid nitrogen. All samples were marked with their treatment group, replicate and sampling date and stored at -20°C. Plasma samples were used for Se assay, while serum samples were detected for antibody levels and antioxidant status. Frozen tissues were also prepared for the determination of antioxidant status.

### Biochemical determinations

The GSH-Px, total antioxidant capability (T-AOC), malondialdehyde (MDA), immunoglobulin A (IgA), immunoglobulin M (IgM), and immunoglobulin G (IgG) levels were determined using commercial assay kits purchased from Nanjing Jiancheng Institute of Bioengineering (Jiangsu, China) following the standard procedures described by the manufacture. GSH-Px can promote hydrogen peroxide react with glutathione, generate H_2_O and oxidized glutathione. The activity of GSH-Px can be reflected by the consumption rate of glutathione. T-AOC was detected by the reduction of Fe^3+^ to Fe^2+^. Fe^2+^ can combine with other substances to form colored clathrate. The condensation of MDA with thiobarbituric acid synthesized red products. GSH-Px, T-AOC and MDA were all determined using colorimetric assay and the results were obtained as optical density using Hitachi 7160 automatic biochemical analyzer (Japan). The GSH-Px and T-AOC levels were expressed as units per milliliter (U/mL) in serum and units per milligram (U/mg) of total protein (TP) in tissues, respectively. MDA concentration was expressed as nanomole per milliliter (nmol/mL) in serum and nanomole per milligram (nmol/mg) of TP in tissues, respectively.

Total level of nonspecific IgA, IgM, and IgG in serum were determined by turbidimetry and expressed as grams per liter (g/L). Tissue TP concentrations were tested by commercial kits from Zhongsheng Beikong Biotechnology and Science Inc. (Beijing, China). The instrument used was Hitachi 7160 automatic biochemical analyzer (Japan).

The detection method of Se content was recommended by NOVUS Intl. [25]. The method is based of two methods, AOAC 2001.19 and a microwave digestion method for selenium. The method utilizes the microwave digestion with nitric acid and hydrogen peroxide. The ramp time is 15 min, hold time is 20 min and cool down time is 15 min with the temperature of 200°C. The samples are diluted into the linear calibration range with 1% methanol in water and analyzed on the ICP-MS (inductively coupled plasma source mass spectrometer). The canonical plotting standard from the ICP-MS is normalized with a rhodium internal standard. The ICP-MS instrument was Agilent 7500 (Santa Clara, CA, USA).

### Statistical analysis

The experimental data were analyzed as a randomized complete block design by using the GLM (General Linear Model) procedures of SAS 9.2 (SAS Inst. Inc., Cary, NC). Blocks were based on initial body weight. Each pen was considered the experimental unit. Pens were considered as random effects and different diets were fixed effect. For antioxidant status, plasma selenium and immune function indicators, which were repeatedly measured at two sampling periods, a repeat measure analysis of variance was employed. If significant differences were found, the Student Newman Keul’s test was used to test the significance of differences between means. A significant level of *P* < 0.05 was applied.

## Results

### Growth performance

During 22-42 d of the experiment, F:G of 0.3 and 0.7 mg/kg DL-SeMet supplementation groups were significantly lower compared with the low-Se group (*P* < 0.05; Table 2). Other parameters were not affected during the periods of 0-21 d, 22-42 d, and 0-42 d.

**Table 2:** **Effect of dietary DL-SeMet supplementation on growth performance of weaning pigs**

Items ^1^	Low-Se	Sodium selenite	DL-SeMet				SEM	***P***-value
	< 0.1	0.3	0.1	0.3	0.5	0.7		
0 d BW, g	9.92	9.93	9.96	9.94	9.96	9.95	0.02	0.39
21 d BW, g	16.33	16.68	16.72	16.16	16.28	16.78	0.16	0.05
42 d BW, g	24.69	26.71	26.07	26.26	25.27	26.76	0.50	0.05
0–21 d								
ADFI, kg	0.46	0.48	0.46	0.44	0.45	0.48	0.02	0.36
ADG, kg	0.30	0.32	0.32	0.30	0.30	0.32	0.01	0.05
F:G	1.49	1.48	1.43	1.46	1.50	1.47	0.03	0.60
Mortality, %	0.00	0.00	0.00	2.78	0.00	2.78	1.43	0.44
22–42 d								
ADFI, kg	0.97	1.06	1.00	1.04	1.01	1.02	0.03	0.44
ADG, kg	0.40	0.48	0.45	0.48	0.43	0.47	0.02	0.05
F:G	2.43^a^	2.24^ab^	2.24^ab^	2.17^b^	2.37^ab^	2.15^b^	0.06	0.01
Mortality, %	0.00	0.00	0.00	0.00	0.00	2.78	1.13	0.44
0–42 d								
ADFI, kg	0.71	0.77	0.73	0.74	0.73	0.75	0.02	0.58
ADG, kg	0.35	0.40	0.38	0.39	0.36	0.40	0.01	0.05
F:G	2.02	1.93	1.90	1.90	2.00	1.87	0.04	0.05
Mortality, %	0.00	0.00	0.00	2.78	0.00	5.56	2.32	0.44

^a-c^Means followed by different letters in a row differ significantly (*P* < 0.05), followed by no or same letters indicated no significant difference (*P* > 0.05).

^1^BW: body weight, ADFI: average daily feed intake, ADG: average daily gain, F:G: feed: gain ratio.

### Antioxidant Status

At the end of the experiment, serum GSH-Px content of 0.3 mg/kg DL-SeMet treatment was significantly higher than the low-Se and 0.3 mg/kg sodium selenite groups (*P* < 0.05; Table 3). The 0.5 and 0.7 mg/kg DL-SeMet groups had higher levels of serum GSH-Px compared with the 0.3 mg/kg sodium selenite group (*P* < 0.05). The serum GSH-Px activity of 0.1 mg/kg DL-SeMet and 0.3 mg/kg sodium selenite supplementation groups were at the same level with the low-Se group. The T-AOC of serum from 0.3 mg/kg DL-SeMet treatment was significantly higher compared with the low-Se and 0.3 mg/kg sodium selenite groups (*P* < 0.05). Serum MDA levels among all treatments were not affected.

**Table 3:** **Effect of dietary DL-SeMet supplementation on serum, liver, and muscle antioxidant status of weaning pigs**

Items ^1^	Low-Se	Sodium selenite	DL-SeMet				SEM	***P***-value
	< 0.1	0.3	0.1	0.3	0.5	0.7		
Serum								
GSH-Px, U/mL	787.52^bc^	774.43^c^	837.67^abc^	892.12^a^	865.23^ab^	859.43^ab^	21.40	< 0.01
T-AOC, U/mL	8.56^b^	8.43^b^	9.19^ab^	9.87^a^	9.23^ab^	9.35^ab^	0.27	0.01
MDA, nmol/mL	3.74	3.36	3.25	3.97	3.96	3.98	0.37	0.58
Liver								
GSH-Px, U/mL	54.50^c^	74.60^ab^	81.61^a^	59.15^bc^	61.02^bc^	66.02^bc^	4.47	< 0.01
T-AOC, U/mL	0.40^b^	0.43^b^	0.63^a^	0.44^b^	0.38^b^	0.46^b^	0.05	0.02
MDA, nmol/mL	1.45^a^	1.04^b^	0.84^b^	0.82^b^	0.86^b^	1.08^b^	0.07	< 0.01
Muscle								
GSH-Px, U/mL	90.05^c^	100.81^c^	109.89^abc^	132.30^a^	129.19^ab^	105.11^bc^	7.07	< 0.01
T-AOC, U/mL	0.85	0.77	0.83	0.73	0.74	0.80	0.07	0.73
MDA, nmol/mL	0.16^a^	0.10^b^	0.12^b^	0.11^b^	0.11^b^	0.11^b^	0.01	< 0.01

^a-c^Means followed by different letters in a row differ significantly (*P* < 0.05), followed by no or same letters indicated no significant difference (*P* > 0.05).

^1^GSH-Px: glutathione peroxidase, T-AOC: total antioxidative capacity, MDA: methane dicarboxylic aldehyde.

Regarding the liver oxidation resistance on d 42, significant responses were observed in GSH-Px, T-AOC, and MDA levels (*P* < 0.05; Table 3). Pigs supplemented with 0.1 mg/kg of DL-SeMet or 0.3 mg/kg of sodium selenite showed increased GSH-Px activity compared with low-Se group (*P* < 0.05). The treatment supplemented with 0.1 mg/kg of DL-SeMet showed the highest T-AOC compared with other treatments (*P* < 0.05). Compared to the low-Se group, the supplementation of both DL-SeMet and sodium selenite significantly reduced the MDA concentration (*P* < 0.05).

Dietary supplementing of different levels of DL-SeMet had effects on GSH-Px activity and MDA concentration in muscles (Table 3). Pigs supplemented with 0.3 and 0.5 mg/kg DL-SeMet had higher content of GSH-Px compared with the low-Se and 0.3 mg/kg sodium selenite groups (*P* < 0.05). Similar to the result of livers, significant reduction (*P* < 0.05) was also found in muscle MDA concentration between the Se supplemented groups and the low-Se group. However, DL-SeMet did not affect the T-AOC across all treatments in muscles.

### Plasma Se concentration

On d 21, pigs supplemented with 0.5 mg/kg DL-SeMet showed significant higher plasma Se concentration compared with the low-Se, 0.3 mg/kg sodium selenite, and 0.1-0.3 mg/kg DL-SeMet groups (*P* < 0.05, Table 4). But there’s no difference between pigs supplemented with 0.5 and 0.7 mg/kg of DL-SeMet. On d 42, plasma Se content increased as the supplemental DL-SeMet increased. Adding 0.3-0.5 mg/kg of DL-SeMet had significant effects on plasma Se content compared with the low-Se group (*P* < 0.05). Plasma Se concentration was much higher than the low-Se and the 0.3 mg/kg sodium selenite supplemented groups when the level of DL-SeMet reached 0.7 mg/kg (*P* < 0.05).

**Table 4:** **Effect of dietary DL-SeMet supplementation on plasma Se content of weaning pigs**

Items ^1^	Low-Se	Sodium selenite	DL-SeMet				SEM	***P***-value
	< 0.1	0.3	0.1	0.3	0.5	0.7		
21 d								
Se, mg/L	0.09^c^	0.13^bc^	0.10^c^	0.13^bc^	0.19^a^	0.17^ab^	0.01	< 0.01
42 d								
Se, mg/L	0.09^c^	0.12^bc^	0.14^abc^	0.17^ab^	0.18^ab^	0.20^a^	0.02	< 0.01

^a-c^Means followed by different letters in a row differ significantly (*P* < 0.05), followed by no or same letters indicated no significant difference (*P* > 0.05).

^1^Se: selenium.

### Immune function

The effect of dietary supplementation of DL-SeMet on humoral immunity level in weaning pigs was shown in Table 5. On d 21 and 42, dietary treatments of DL-SeMet and sodium selenite had no effect on serum IgA, IgM, and IgG levels.

**Table 5:** **Effect of dietary DL-SeMet supplementation on antibody levels of weaning pigs**

Items ^1^	Low-Se	Sodium selenite	DL-SeMet				SEM	***P***-value ^*^
	< 0.1	0.3	0.1	0.3	0.5	0.7		
21 d								
IgA, g/L	1.17	1.07	1.14	1.11	1.25	1.00	0.07	0.30
IgM, g/L	0.89	0.81	0.81	0.85	0.88	0.75	0.04	0.15
IgG, g/L	8.60	8.16	8.46	8.81	9.14	7.94	0.34	0.19
42 d								
IgA, g/L	1.07	1.05	1.09	1.16	1.14	1.10	0.07	0.85
IgM, g/L	0.88	0.78	0.84	0.91	0.88	0.94	0.05	0.30
IgG, g/L	8.70	8.52	8.54	8.74	8.78	8.99	0.27	0.85

^*^Means followed by different letters in a row differ significantly (*P* < 0.05), followed by no or same letters indicated no significant difference (*P* > 0.05).

^1^IgA: immune globulin A, IgM: immune globulin M, IgG: immune globulin G.

## Discussion

### Growth performance

The effects of Se on growth performance of animals are somewhat variable. In the present study, pigs fed with 0.3 and 0.7 mg/kg of DL-SeMet had significantly lower F:G during d 22-42. DL-SeMet could slightly improve the growth performance of weaning pigs compared with the low-Se group. Jiang et al. [15] made a conclusion that 0.225 mg/kg of selenomethionine could highly improve the growth performance of broilers. Zhan et al. [14] reported that maternal selenomethionine intake of 0.3 mg/kg significantly increased the average weight gain of piglets from birth to weaning compared with the 0.3 mg/kg sodium selenite group. While Miller et al. [26] reported no difference in performance of broilers fed with various levels (0-0.5 mg/kg) of Se from different Se sources (sodium selenite or selenomethionine). Even some controversial conclusions demonstrated that neither Se source nor Se level had any effect on growing-finishing pigs and broilers [8, 27, 28]. The inconsistent conclusions may cause by the difference of organic Se used in these researches. Breeds or stages of experimental animals may also affect the results. In addition, tocopherol in diet can protect animals from oxidative damage [29], weakening the effect of Se deficiency. Besides, all pigs in our experiments were previously fed with the basal diet containing 0.06 mg/kg Se for one week, the Se stored in tissues were consumed. So the carryover effect of tissue Se from nursery to weaning was decreased and last for a relatively short duration, which might lead to the only remarkable difference among groups during d 22-42.

### Antioxidant status

Se is an integral part of GSH-Px, which is a kind of antioxidant enzyme that helps to control the levels of hydrogen peroxide and lipid peroxides produced during normal metabolic activities [30]. In our study, the highest serum and muscle GSH-Px activity was found in treatment fed with 0.3 mg/kg of DL-SeMet compared with the low-Se and the 0.3 mg/kg sodium selenite groups. The addition of 0.1 mg/kg DL-SeMet mostly improved the level of GSH-Px in liver. DL-SeMet was more effective than sodium selenite. Similar results were reported by Zhan et al. [6, 14], while Mahan et al. [7, 27] demonstrated that sodium selenite had a higher bioavailability than organic Se-enriched yeast in meeting the need of this selenoprotein in growing and finishing pigs.

It was widely accepted that proper Se intake could enhance the antioxidant ability of the body. T-AOC indicates the oxidation resistance capacity of the whole body. MDA is one of the final products of polyunsaturated fatty acid peroxidation in cells and is considered as a marker of oxidative stress [31]. According to our results, the highest serum T-AOC level was also observed in 0.3 mg/kg DL-SeMet supplemented group compared with the low-Se and the 0.3 mg/kg sodium selenite groups. While Se supplementation had no effect on muscle T-AOC, 0.1 mg/kg of DL-SeMet enhanced liver T-AOC mostly. Furthermore, tissue MDA concentration was lower in all Se supplemented groups. Zhan et al. [6] reported that supplement 0.3 mg/kg of Se to the diet of finishing pig significantly decreased the content of MDA both in liver and muscle, the content of tissue MDA in selenomethionine treated group was slightly lower than that in sodium selenite group, especially in muscle. Wang et al. [32] pointed out that 0.15 mg/kg of selenomethionine supplementation was more effective than sodium selenite to increase T-AOC and decrease MDA concentration in serum and organs.

Above findings suggested that dietary supplementation of Se could improve physical antioxidant ability. Diets supplementation with 0.1-0.3 mg/kg of DL-SeMet seemed to be more effective and advantageous in improving the oxidation resistance of weaning pigs.

### Plasma Se concentration

Dietary Se supplementation obviously increased plasma Se content and, to some extent, plasma Se content reflected the concentration of Se supplemented to the diet. Similar results were reported by Mahan et al. [7], who evaluated the efficacy of inorganic and organic Se fed to growing-finishing pigs. They pointed out that serum Se concentrations increased (*P* < 0.01) as the dietary level of Se increased. Serum Se was somewhat higher at the 0.05 mg/kg supplemental Se level when sodium selenite was the Se source, but, as the dietary Se level increased, serum Se concentrations were higher when organic Se source was provided. Kim and Mahan [19] subsequently found that, compared with sodium selenite, the plasma Se concentration approximately doubled when Se-enriched yeast was fed.

The difference of accumulation between inorganic and organic Se might be explained by the differences of metabolic pathways. Se in both forms can be incorporated into GSH-Px, but selenomethionine might also be incorporated in other proteins in substitution for methionine [33, 34]. Selenomethionine could be effectively metabolized to a selenium analogue of *S*-adenosylmethionine, and was further metabolized in transmethylation reactions and in polyamine synthesis, similarly to the corresponding sulphur metabolites of methionine [35].

### Immune function

Adequate intake of Se is required to ensure optimal immune function. Various components of the immune system fail to function correctly if dietary Se is deficient [36]. Peplowski et al. indicated that Se provided in diet or by injection increased the immune response of weaning pigs after an antigenic challenge with sheep red blood cells [37]. It was demonstrated that the combined supplementation of Se and vitamin E was particularly effective in raising the antibody responses of animals which were deficient in both nutrients [37–39], but was less effective in animals which were already receiving adequate levels of one or both nutrients [40, 41]. No differences were found in IgA, IgM, and IgG levels across all treatment groups on d 21 and 42 in our study, which might due to the supplementation of vitamin E in the diet. In addition, pigs in this experiment were not infected by external antigens.

## Conclusions

The present study demonstrated that DL-SeMet can replace sodium selenite in the diet of weaning pigs. The supplementation of DL-SeMet in weaning pig diet could improve growth performance, antioxidant status, and plasma Se content at the level of 0.1-0.7 mg/kg. The optimum level of DL-SeMet is suggested to be 0.1-0.3 mg/kg, and the addition of 0.7 mg/kg DL-SeMet had no adverse effect on weaning piglets.
